# Evaluation of Lapatinib-Loaded Microfibers Prepared by Centrifugal Spinning

**DOI:** 10.3390/polym14245557

**Published:** 2022-12-19

**Authors:** Enikő Bitay, Attila Levente Gergely, József Kántor, Zoltán-István Szabó

**Affiliations:** 1 Department of Mechanical Engineering, Faculty of Technical and Human Sciences, Sapientia Hungarian University of Transylvania, Târgu-Mureş, Târgu-Mureş/Corunca, Calea Sighișoarei nr. 2., 540485 Târgu-Mureş, Romania; 2 Research Institute of the Transylvanian Museum Society, 2–4 Napoca, 400009 Cluj, Romania; 3 Department of Drugs Industry and Pharmaceutical Management, George Emil Palade University of Medicine, Pharmacy, Science, and Technology of Targu Mures, Gh. Marinescu 38, 540485 Târgu-Mureş, Romania; 4 Sz-imfidum Ltd., 525401 Lunga, Romania

**Keywords:** lapatinib, centrifugal spinning, fiber mat, amorphous solid dispersion, scale-up

## Abstract

Lapatinib (Lap) is a lypophilic drug frequently used in cancer treatment; however, due to its limited solubility and permeability, achieving therapeutic dose through oral administration proves to be a challenge. There are various methods for enhancing the solubility of Lap and other similar drugs, one being the preparation of amorphous solid dispersions (ASD). In this study, a Lap-loaded polyvinylpyrrolidone (PVP) fiber mat was created with centrifugal spinning from a PVP/Lap solution in dimethyl formamide and ethanol. The production rate was 12.2 g/h dry fibers, and the fibers had an average thickness of 2.55 ± 0.92 μm. In the differential scanning calorimetry (DSC) thermogram of the fiber mat, the melting peak of the crystalline Lap was not visible, suggesting that Lap was in an amorphous state. A dissolution study was carried out in 0.2 M phosphate buffer saline solution at 37 °C. UV spectrophotometry data indicated that in the sample containing the fiber mat, the Lap concentration was 332 μg/mL (66%) in 10 min, decreasing to 227 μg/mL by 45 min. Meanwhile the crystalline Lap formed a 30–40 μg/mL (6–8%) solution in 5 min, maintaining that concentration. We conclude that centrifugal spinning can be an effective and easy method to produce ASDs.

## 1. Introduction

Lapatinib (Lap) is a synthetic molecule with a quinazolin-4-amine precursor that has a molar mass of 581.06 g/mol and is highly lypophilic (logP = 5.49). The potent tyrosine kinase inhibitor is currently used in combination with capecitabine or letrozole for advanced or metastatic HER2+ breast cancer [1,2]. Considering its high lypophilicity, it easily passes through the blood–brain barrier, and for this reason, it also has a potential application in the treatment of metastatic brain tumors [3]. At the same time, the lypophilicity of Lap is a drawback when it comes to oral administration. The pharmacokinetics properties of the drug are far from ideal, as patients are required to take 5–6 film-coated tablets, due to its low absorption, which is hindered by the low, pH-dependent solubility of the drug. The oral bioavailability of the drug is thus largely affected by meals and gastric pH, which can lead to suboptimal plasma concentrations of Lap, while large doses can lead to non-adherence to therapy [2,4,5,6,7].

Multiple methods have been developed for the improvement of the solubility of Lap in aqueous media. Physical methods include particle size reduction, solubilization by cosolvency or hydrotropy, recrystallization by ultrasound (sonocrystallization) or supercritical fluid process, and the preparation of amorphous solid dispersions (ASD) [8,9,10,11]. There are also chemical methods such as functionalization and prodrug formation [10].

The ASD method involves the embedding of the drug into a polymeric matrix, which will inhibit aggregation of the drug, forcing it into an amorphous state. Because the amorphous drug is in a higher state of energy compared to its crystalline form, it dissolves more readily [12]. The choice of polymer as the stabilizing matrix is important, as the functional groups determine how well the drug interacts with it. Both ionic interactions and hydrogen bonding have been proposed as a stabilizing mechanism. De Araujo et al. studied the bonding of Lap with hydroxypropyl methylcellulose phthalate (HPMCP) and hydroxypropyl methylcellulose (HPMC-E3) using High-Energy X-ray Diffraction (XRD) and differential pair distribution function analysis. The results indicated that the Lap was dispersed well in the HPMCP (attributed to the reaction between the carboxyl groups of the phthalate and the NH groups of the Lap), but the HPMC-E3 sample showed significant drug–drug interaction [13]. Song et al. performed glass transition temperature (T_g_) measurement on various Lap-ASDs produced with spray drying and concluded that hydroxypropyl methylcellulose acetate succinate (HPMCAS) interacted with Lap through hydrogen bonds, while HPMCP likely participated in acid–base reaction with Lap, also supported by ^15^N nuclear magnetic resonance (NMR) spectroscopy data [14].

Song et al. also made two other ASDs of Lap (40% w/w) with Soluplus^®^ and poly vinylpyrrolidone vinyl acetate (PVPVA), in addition to the previously mentioned HPMCAS and HPMCP, and studied the Lap dissolution from these. The results indicated that in sodium dodecyl sulfate (SDS) medium with a concentration of 2 mg/mL and at 37 °C, the dissolution of Lap increased from ~5% for the pure Lap to 65–75% for the ASDs, except for the PVPVA, in which case 20% of the Lap was dissolved after 2 h. Initially, the Soluplus^®^ released the most Lap, but the concentration started decreasing after 15 min. By the 2 h mark, the HPMCAS sample had the most Lap dissolved, at ~75% [14].

Hu et al. prepared ASDs of Lap in Soluplus^®^ and Poloxamer 188 mixture with two different methods, solvent rotary evaporation (SRE) and hot melt extrusion (HME); 5% w/w KHCO_3_ was included in the formulations to prevent gel formation. DSC and XRD data suggested that the HME samples contained crystalline Lap, while no such traces were found in the case of the SRE samples. The dissolution study was done in 0.1 M HCl solution with 2% polysorbate 80 at 37 °C. After 60 min, ~92% of the Lap was released from the SRE sample, while ~67% of the crystalline Lap was dissolved. The HME sample produced higher dissolution values than the pure Lap; however, the authors concluded that the difference was not statistically significant [15].

Prabhu et al. developed Lap-containing nanosponges (NSs) made from a copolymer of poly(ethyl acrylate), poly(methyl methacrylate), and poly(chlorotrimethyl ammonioethyl methacrylate), available under the trade name of Eudragit RS100, and polyvinyl alcohol (PVA). The NS fabrication involved a successive mixing of Eudragit and Lap EtOH solution with aqueous PVA solution, continuously stirring at 40°C until the EtOH evaporated, followed by freeze-drying to remove the water and acquire a fine powder. Both DSC and XRD data indicated greatly reduced crystallinity of the Lap in the NSs. Solubility studies showed that in a 6 h span at pH 6.8, nearly 100% of the Lap from the NSs was dissolved, as opposed to the 5% of the pure Lap, and the NSs provided three times higher plasma concentration in vivo 4 h after administration [16].

In addition to the aforementioned procedures, electrospinning, a popular nanofiber production method, has also been used to produce nanofiber-based ASDs [17,18]. During electrospinning, an electrically charged polymer solution is dispensed through a nozzle. At the tip of the nozzle, a pendent drop forms, and the generated electric field (50–500 V/mm) distorts its shape, forming the Taylor cone. Upon reaching critical charge density, a liquid jet emerges from the tip of the Taylor cone and travels towards the grounded electrode (referred to as the collector), during which it becomes elongated by Coulomb forces [19]. Over time, the solvent evaporates from the jet, the local charge density increases, and the jet becomes unstable, assuming a coiled shape. This phenomenon is called the bending instability, and it is pivotal in the formation of nanofibers. Fibers as thin as 50 nm in diameter can be produced with electrospinning [19].

Earlier, our group published a report on the fabrication of ASDs of Lap and PVP with electrospinning. The fibers had an average diameter of 461 ± 160 nm, and Lap dissolution from the ASD in 0.2 M PBS reached ~70% in 3 min, as opposed to the 5% dissolution of the pure Lap [20]. This was the first report of Lap-containing nanofiber-based ASDs. The results indicate the viability of nanofiber-based ASDs when compared to the other ASD production methods cited above. The production of nanofibers with electrospinning is a widely used technique due to is simplicity, flexible setup, and low upfront cost. There are several methods to increase the productivity of the electrospinning process [21,22,23,24], all of which involve the use of a large polymer solution surface. These methods are known as needleless electrospinning, since no needle is involved in the formation of the Taylor cones; instead, a large metal surface is wetted with the polymer solution, or the Taylor cones form directly on the free liquid surface. The polymer solution concentration and composition changes during needleless electrospinning due to solvent evaporation and potential moisture absorption. Although the productivity is increased, and there is a commercially available machine, NanoSpider^™^, the use of high potential difference (>50 kV) and the electrical conductivity requirement of the polymer solution remain disadvantages. The electrical conductivity of intrinsically nonconductive electrospinning solutions is increased by additives [16,17], which make the system more complex, potentially inhibit certain applications (e.g., if they are toxic), and add extra cost.

**Figure 1:**
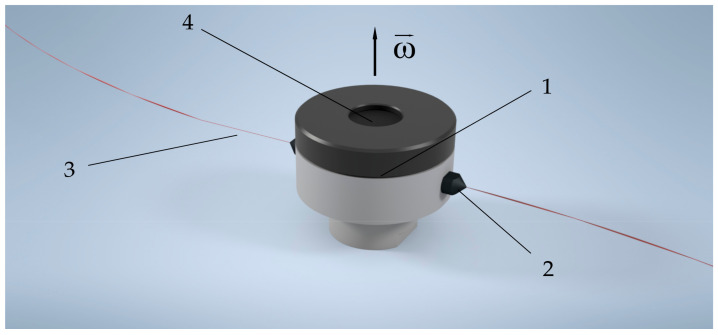
Basic centrifugal spinning setup. 1—spinner head; 2—nozzle; 3—polymer solution jet; 4—solution reservoir.

Centrifugal spinning is another means to produce non-woven polymer fiber mats. The central component of centrifugal spinning equipment is a rotating chamber with radially placed nozzles. A scheme of a centrifugal spinning head is shown in Figure 1.

The chamber or spinner head is rotating at a speed of 3000–15,000 rpm, and the solution inside the chamber is expelled through the nozzles. As the polymer solution passes through the nozzle, a jet is formed, and the jet becomes elongated by the centrifugal force and, in some part, by the Coriolis force. During the elongation, the solvent evaporates from the jet, resulting in the formation of dry fibers. The fibers are then caught by a screen or some other obstacles placed around the spinner head, called the collector. The fiber generation can be maintained as long as solution is being fed into the chamber. A significant difference between centrifugal spinning and electrospinning is that centrifugal spinning does not require a high voltage source (although it does require a motor), but more importantly, the used polymer solutions do not need to be electrically conductive. This provides more flexibility, with the choice of polymer/solvent systems and solution formulations, especially in case of non-polar polymers.

Studies show that, in general, there is little difference between the size of the fibers acquired by centrifugal spinning and electrospinning, except for the sub-100 nm range [25]. The most noticeable advantage centrifugal spinning has over electrospinning is its greatly increased productivity, at least as far as rudimentary laboratory setups are concerned. The typical electrospinning setup contains a single nozzle, and volumetric flow rates are in the 1–3 mL/h range (0.1–1 g/h dry fibers) [26,27], while dual-nozzle centrifugal spinning setups can achieve production rates around 20–60 g/h dry fibers under laboratory conditions [28,29]. The needleless electrospinning setups also have a higher throughput than the single nozzle setups, comparable even to centrifugal spinning in some cases [23,24,27,30]; however, these require \more specialized equipment and have the specified limitations.

The use of centrifugal spinning in ASD production is limited. Marano et al. reported on the centrifugal spinning of olanzapine (OLZ) and piroxicam (PRX)-loaded sucrose fibers; however, they performed the spinning using melt instead of solution. The fiber sizes ranged from 10 ± 3 μm to 14 ± 4.5 μm. Dissolution studies in PBS showed that the drugs released from the ASD reached 3 and 1.4 times higher concentrations in 4 h than the pure OLZ and PRX, respectively [31]. The performance of the ASDs remained virtually identical after 8 months of aging [32].

The aim of the work is to examine the applicability of the centrifugal spinning to produce drug-loaded nanofibers. The model drug used in this study is Lap, and PVP is used as the carrier. The first step is the fabrication of the fiber mat with the Lap in it, followed by analyses to determine whether the formation of an ASD was successful. The solubility of the Lap loaded into the centrifugally spun mat is then tested and compared to the solubility of pure crystalline Lap and to that of the Lap from the electrospun fiber mat. The end goal is to have a simple, inexpensive, and easy method to produce well-performing drug carriers; centrifugal spinning has the prospect to fit in that role.

## 2. Materials and Methods

### 2.1. Materials

Polyvinylpyrrolidone (PVP, Sigma Aldrich, K90, M_w_ = 360.000 g/mol), ethanol (EtOH, VWR, 99.9%), and *N*,*N*-dimethylformamide (DMF, Alfa Aesar, ACS grade) were used as received. Lapatinib (Lap) as a ditosylate monohydrate salt was obtained as a gift sample from a local pharmaceutical company in Targu Mures, Romania.

### 2.2. Solution Preparation

For the scouting experiments, 15, 19, and 22% w/w PVP solutions in 50/50 v/v DMF/EtOH solutions were prepared.

The composition of the solution used for the production of the drug-loaded fiber mat was 22% w/w PVP (750 mg), 3% w/w Lap (100 mg), 41% w/w DMF (1.5 mL), and 34% w/w EtOH (1.5 mL). The solution was prepared by dissolving 100 mg Lap in 1.5 mL DMF, resulting in a yellow solution, followed by the addition of 1.5 mL EtOH, and 750 mg PVP. The PVP was dissolved in 25 min at room temperature at a stirring speed of 500 rpm.

### 2.3. Centrifugal Spinning

The centrifugal spinning setup was based on the results obtained by Sebe et al. regarding laboratory-scale high-speed rotary devices [33]. Centrifugal spinning was carried out in the range of 3500–7500 rpm during the scouting experiments, with a collector of 200 mm in diameter and a 0.3 mm capillary inner diameter (ID). The collector consisted of 14 metal rods placed around the spinning head at equal distance. The Lap-containing fiber mat was spun at 5500 rpm (based on the results obtained from the scouting experiments), with all other parameters being identical with those of the scouting experiments. All experiments were carried at room temperature (~20 °C) and ~50% relative humidity.

### 2.4. Scanning Electron Microscopy (SEM)

SEM imaging was performed with a JEOL JSM-5200 scanning electron microscope at 15 kV acceleration voltage on uncoated samples. ImageJ was used to measure the fiber sizes, and 100 measurements were taken on each sample.

### 2.5. Measurement of Lap Content in the Fiber Mats

Three samples were taken from the produced Lap-containing fiber mat and dissolved in DMF. The solutions were placed into 1 mL plastic cuvettes. The Lap content of the samples was measured with UV-Vis spectrophotometry using a Shimadzu UV-1601PC spectrophotometer. The spectra were recorded at the 260–450 nm range. For reference, a 0.12 mg/mL Lap solution in DMF was used.

### 2.6. In Vitro Dissolution Study

The release of Lap was studied in 0.2 M phosphate buffer solution at a pH of 6.8. Two fiber mat samples were placed in separate vials containing 10 mL buffer. Two additional samples were made with pure Lap only. Each sample contained 5 mg Lap, and equivalent Lap containing fiber mats. During dissolution, the samples were kept at a temperature of 37 ± 0.1 °C and were constantly stirred at 400 rpm with a magnetic stirrer. At 5, 10, 15, 30, and 45 min, aliquots of 0.5 mL were taken from the samples and filtered through 0.2 μm regenerated cellulose filters into 1 mL plastic cuvettes.

The released Lap amount in the aliquots was determined with UV spectrophotometry based on the absorbance at 262 nm wavelength using a Shimadzu UV-1601PC spectrophotometer. Pure buffer solution was used as baseline for the measurements. Some aliquots needed to be diluted with buffer solution, and this was done directly in the cuvettes.

### 2.7. Differential Scanning Calorimetry (DSC)

DSC measurements were performed with a Shimadzu DSC-60 calorimeter. Samples of 5–10 mg were placed into aluminum pans, and the pans were sealed. An empty aluminum pan of the same type was used as reference. The process consisted of a heat cycle in the temperature range of 30 to 300 °C at a 10 °C/min heating rate.

## 3. Results and Discussion

### 3.1. Polymer Fiber Production

The production of nanofiber-based Lap solid dispersion using electrospinning has been reported [20]. In our previous work, we established the solution composition of 15% w/w PVP and 3% w/w Lap in 50/50 v/v DMF/EtOH. To compare the dissolution results of electrospun and centrifugal spun fiber-based ASDs, we kept the composition of the ASD similar yet optimized to the fiber production method.

**Figure 2:**
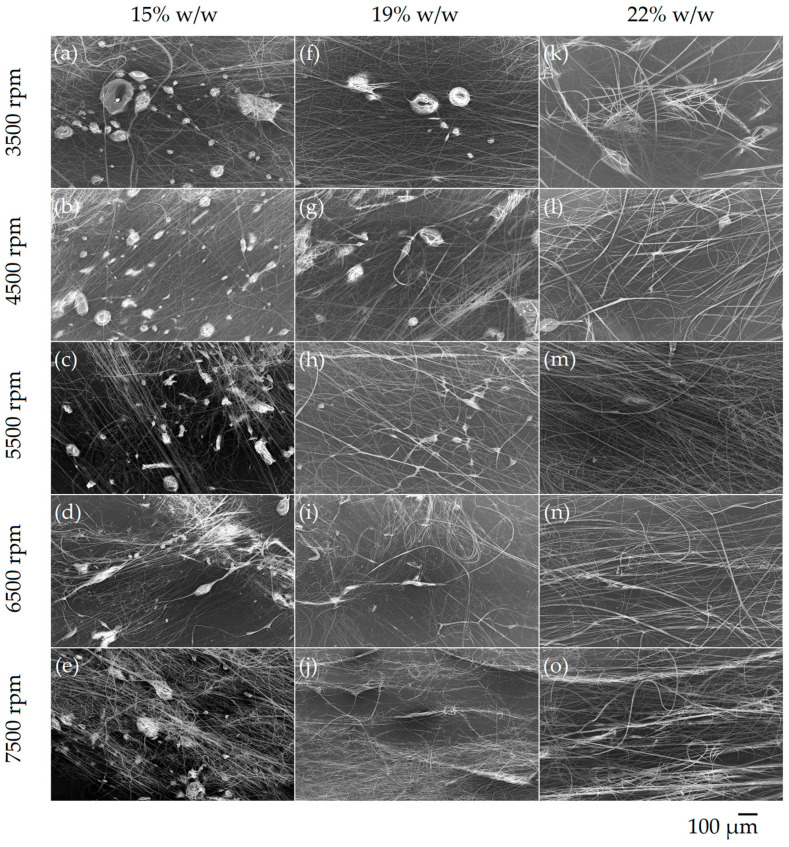
SEM micrographs of PVP fibers spun from 15% w/w (**a**–**e**), 19% w/w (**f**–**j**), and 22% w/w (**k**–**o**) solutions at various speeds. The images were taken at x100 magnification.

In this study, the effect of PVP concentration and rotational speed on the fiber morphology was investigated. An SEM image for each condition can be seen in Figure 2. Both 15 and 19% w/w PVP solutions resulted in beaded fibers at any speed, except for the 19% w/w solution at 7500 rpm. Fibers spun from the 22% w/w solution had beads at 3500–4500 rpm, but beading was not observed at 5500 rpm and above.

**Figure 3:**
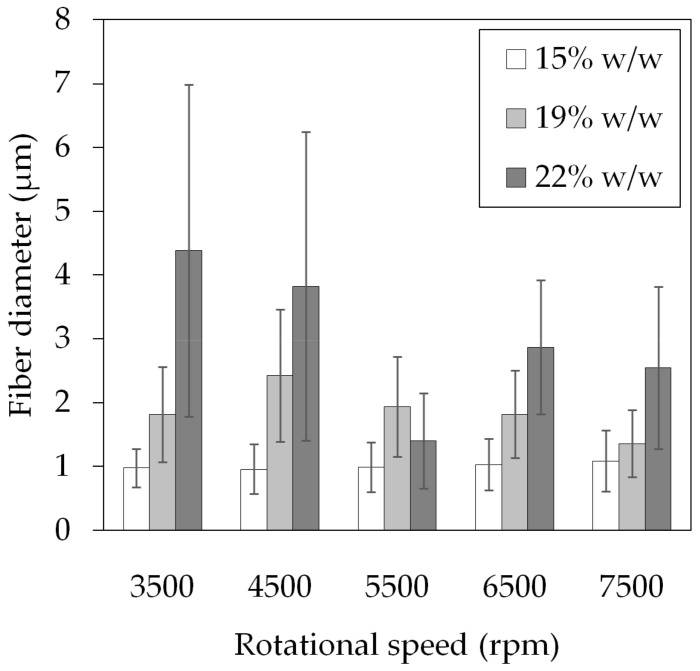
Average fiber size and standard deviation of pure PVP fibers spun at different concentrations and rotational speeds.

The measured fiber sizes are presented in Figure 3. At 15% w/w PVP, both the average fiber diameters and size distributions were consistent at around 1.0 ± 0.4 μm throughout all rotational speeds; however, all samples contained beads. As the PVP concentrations increased, so did the average fiber size, and the beads disappeared. It is known that solutions with low viscosity (caused by low polymer concentration) yield thin and beaded fibers; as the viscosity increases, thicker and smooth fibers are produced [34]. During centrifugal spinning, the beading process is driven by the force balance between the tensile forces (originating from the viscoelastic force and the centrifugal force) and the surface tension. Increasing the viscoelastic and centrifugal forces, which are analogous with the PVP concentration and rotational speed in our case, reduce bead formation. This may be the reason why there were beads in the 22% w/w samples at 3500–4500 rpm but not at higher speeds. The occurrence of thick fiber outliers inflated both the average size and standard deviation.

The prepared PVP/Lap solution was viscous, transparent, and with a yellow color, characteristic of Lap. Table 1 shows the measured fiber sizes of the samples made from 22% w/w PVP solution. The diameter of fibers spun at 5500 rpm had the lowest average value as well as standard deviation, 1.40 ± 0.75 μm, and as such, this condition was chosen for the fabrication of the Lap-loaded PVP fiber mat. In addition, at 6500 rpm and above, the fibers had the tendency to wrap around the spinning head, which was yet another reason to choose the 5500 rpm speed.

**Figure 4:**
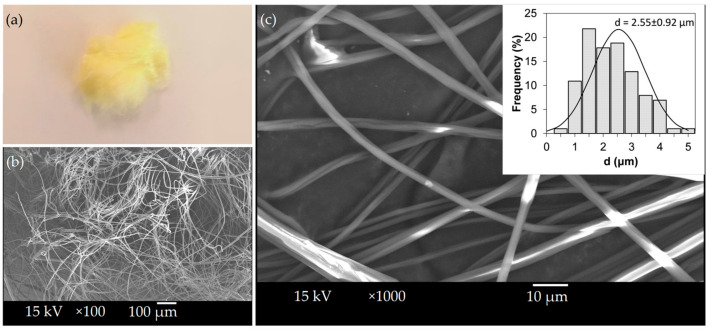
Centrifugally spun Lap-loaded fiber mat. (**a**) Photograph; (**b**) SEM micrograph at ×100 magnification; (**c**) SEM micrograph at ×1000 magnification.

The spinning of Lap-loaded fibers lasted for 100 s, and after drying, 340 mg yellow fibers were acquired (Figure 4a). This extrapolates to a throughput of 12.2 g dry fibers/h. SEM imaging of the Lap-loaded fiber mat showed smooth surface fibers (Figure 4b,c), and although it is not apparent in either micrograph, some of the fibers were beaded. The fibers had an average size of 2.55 ± 0.92 μm, roughly 1.7 times higher than the diameter of the pure PVP fibers under otherwise identical conditions.

### 3.2. Analytical Studies

**Figure 5:**
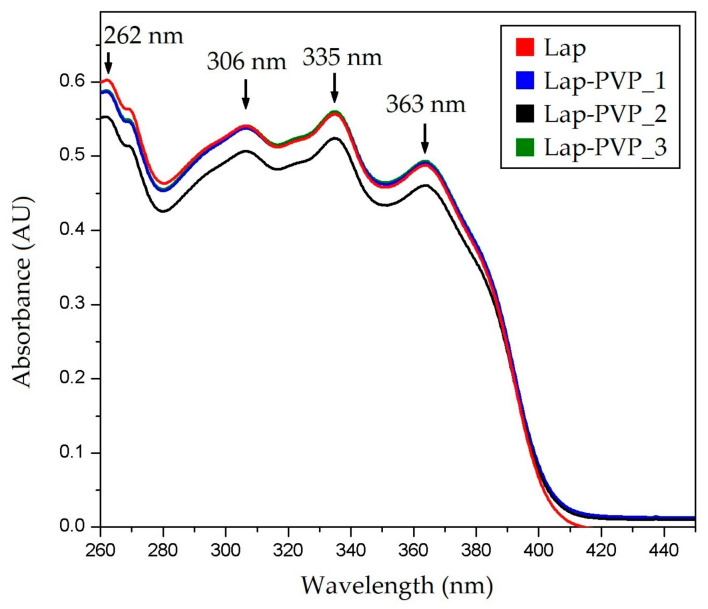
Overlaid representative UV-Vis spectra of the reference solution (Lap) and sample solutions showing the incorporation of the active substance in the prepared microfibers.

Figure 5 shows the UV-Vis spectra acquired from the three samples taken from the Lap-containing fiber mat as well as the pure Lap solution in DMF. The spectra were recorded between 260 and 450 nm wavelength. Four distinct absorbance peaks were observed at 262, 306, 335, and 363 nm for both the Lap and the fiber mat samples, indicating that the fibers do indeed contain Lap. The fiber mat samples were prepared in such a way that roughly the same amount of Lap was present in each sample.

After normalizing the concentrations for the mass of the dry fibers, based on the absorbance read at 262 nm, the Lap content of each sample was acquired, as presented in Table 2.

The theoretical Lap content of the fiber mat would be 11.76% w/w (calculated from the composition of the solution) if the distribution of the Lap were perfectly homogeneous throughout the entirety of the fiber mat. The results show that the distribution of the Lap in the fiber mats was quite homogeneous, with 1–2% difference. The average relative content—compared to the theoretical value—was 101%, which is in the generally acceptable limit for the active substance content (100 ± 5% of the declared content).

**Figure 6:**
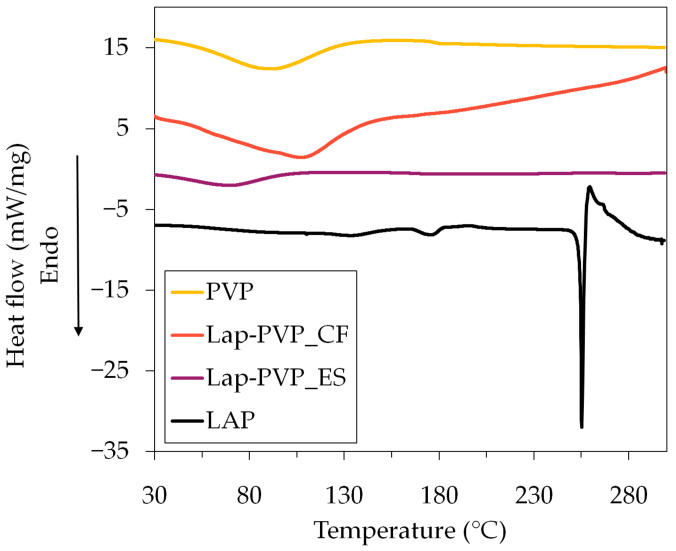
DSC thermograms of the pure Lap, the centrifugally spun Lap-PVP fibers (Lap-PVP_CF), and the electrospun fibers (PVP_ES and Lap-PVP_ES).

The DSC thermograms of the pure compounds and the centrifugally spun Lap-PVP fiber mats can be seen in Figure 6. The previously reported DSC data of the electrospun fiber mats (pure PVP and Lap-PVP) were also included for comparative purposes [20].

The thermogram of all three fiber mat samples contained a broad endothermic peak between 50 and 150 °C, which is likely due to dehydration. At around 178 °C, a step in the thermogram of the PVP_ES sample can be seen, which indicates the glass transition of PVP [35]. Endothermic peaks appeared at 133, 175, and 255 °C in the Lap data. The first two peaks are attributed to the polymorph forms of Lap occurring in small fractions [36], while the largest peak at 255 °C represents the melting of Lap, underlining its crystalline nature. The melting peak of Lap is completely missing from both the Lap-PVP_CF and Lap-PVP_ES, suggesting that in the prepared fiber mats, Lap was not in its crystalline form.

### 3.3. Lap Dissolution

**Figure 7:**
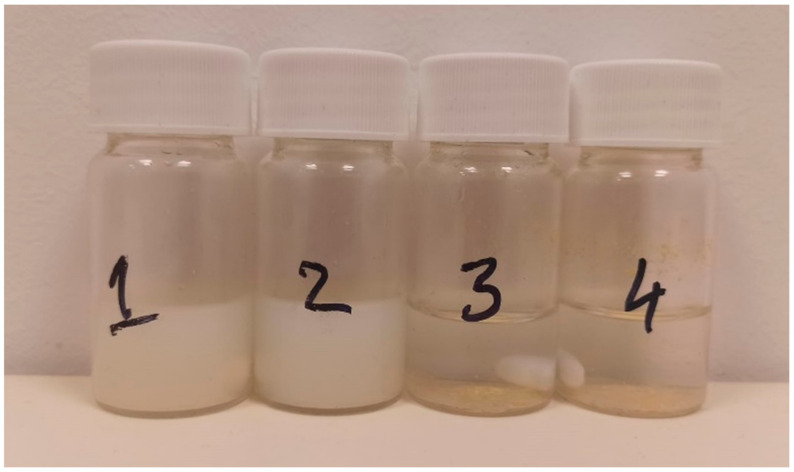
Samples prepared for the dissolution study. Vials 1 and 2 contain the fiber mat, and samples 3 and 4 contain pure Lap. The picture was taken 45 min into the dissolution study.

The fiber mat samples formed an opaque white liquid with the buffer solution as soon as the stirring started and remained that way throughout the dissolution study (samples 1 and 2 in Figure 7.). The samples containing pure Lap remained clear, with yellow particles present at the bottom of the vials, even after 45 min (samples 3 and 4 in Figure 7).

**Figure 8:**
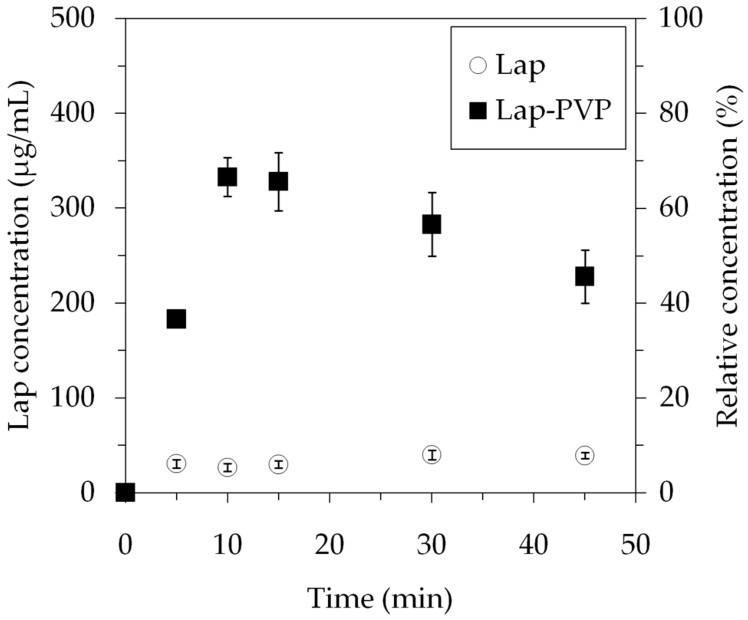
Dissolution profile of Lap-loaded microfibers and pure active substance (average of two measurements ± SD).

The Lap dissolution data is shown in Figure 8.

The graphs indicate that the Lap found in the fiber mat dissolved in the buffer at a much higher percentage than the pure compound. Each sample contained 5 mg Lap and 10 mL buffer, which would have resulted in a concentration of 500 μg/mL if the entire Lap amount had dissolved. In reality, the measured peak concentration for the Lap-PVP was 332 μg/mL, equating to 66% Lap dissolution by 10 min, while the pure Lap samples had a concentration of ~30 μg/mL (6% of the added Lap dissolved). There was a noticeable decrease in the Lap concentration in the case of the Lap-PVP samples after the 10 min mark, with the concentration reaching 227 μg/mL by 45 min. The most likely explanation is that over time the Lap started to recrystallize and precipitated from the solution. There were no visual cues for this, as the samples already contained the disintegrated PVP fiber mat, making the observation of a small amount of additional precipitate impossible. Other research groups also observed this phenomenon while working with similar drug/polymer systems [14,37,38]. It is described as the spring effect, where the amorphous drug supersaturates the solvent, causing a surge in the concentration, followed by a rebound when the drug becomes metastable and crystalline clusters start to form [39].

### 3.4. General Discussion

The data presented in this paper supports the idea that centrifugal spinning can be a viable method for the production of amorphous solid dispersions of drugs.

At this point, direct comparison can be made between centrifugal spinning and electrospinning. We reported earlier that the electrospinning process was stable, with a volumetric flow rate of 0.9 mL/h, which is 0.11 g/h (1.83 mg/min) for dry fibers, if all the generated fibers can be collected [20]. The production rate of the centrifugal spinning was 200 mg/min, measured within an interval of 1.66 min, having the prospect to extrapolate to 12.2 g/h dry fibers. This is a ~110-fold increase in the worst-case scenario. In reality, some of the fibers are lost by being deposited on surfaces adjacent to the fiber collector, so this figure might be even higher.

The fibers produced by electrospinning were 0.462 ± 0.16 μm in diameter, while the centrifugal spinning yielded fibers of 2.55 ± 0.92 μm thickness, a difference by almost an order of magnitude. It is fitting to ask whether the fiber size has any effect on the drug stabilization. Evaluating the performance of the fiber mats, we saw that the electrospun sample reached 70% relative concentration (100% is all the drug being in the dissolved state), declining to 60% at 30 min, while the maximum relative concentration with the centrifugally spun sample was 66%, which decreased to 56% at 30 min and further decreased to 45% by 45 min. Overall, the drop in concentration in the first half-hour in terms of absolute concentration values, 50 μg/mL, was identical for both cases, and the peak values were close. At this point, there is no clear evidence that smaller fiber size better facilitated Lap solubility.

There is very limited data on Lap-PVP ASDs, so a comparison between centrifugal spinning and other ASD production methods is difficult. However, Lap ASDs with other polymers like HPMCP reach ~75% relative concentration and decline much slower [14]. In the case of ketoconazole, both polyacrylic acid and poly(2-hydroxyethyl methacrylate) greatly outperformed PVP [37]. The Lap molecule has two strong hydrogen bond acceptors, the SO_2_ and one of the NH groups [14], and there are no hydrogen bond donors in PVP. Additionally, there are no proton donors for acid-base reaction with the NH groups of the Lap. The NH groups can act as weak-medium strength hydrogen bond donors, so the interaction with the carbonyl groups found in PVP is possible. Still, an argument could be made that switching out PVP to a different polymer would further improve the efficacy of the devices manufactured with centrifugal spinning.

Centrifugal spinning is inherently suitable for producing ASDs, since the fiber formation involves the rapid evaporation of the solvent, which leads to quick solidification of the solute and thus the formation of ASD. It being a single step process means it is less labor intensive or at least comparable to the other methods currently in use.

## 4. Conclusions

In conclusion, Lapatinib-loaded PVP fiber mats were successfully produced with centrifugal spinning for the first time. We found that PVP concentration and rotating speed during centrifugal spinning had a profound effect on the fiber formation process, influencing both fiber size and morphology. At low concentrations, relatively small fiber size, ~1 μm, and beading was observed. Bead formation disappeared at higher concentrations; however, the fiber size increased to a maximum of 4.5 μm at 3500 rpm. The size of the acquired Lap-containing PVP fibers was 2.55 ± 0.92 μm. The DSC and solubility studies indicated that the fiber mats were ASDs of Lap in a PVP matrix. The Lap from the ASD reached 66% dissolution in 5 min, while the dissolution of pure Lap plateaued at ~8%, resulting in an eight-fold increase in the dissolution rate. The productivity of centrifugal spinning was 12.2 g dry fibers/hour, surpassing the electrospinning method, at 0.11 g dry fibers/hour, by more than two orders of magnitude. The Lap dissolution from PVP nanofiber solid dispersions prepared by electrospinning and centrifugal spinning was basically the same, ~70%, indicating that centrifugal spinning is a promising scale-up process that eliminates several shortcomings of the electrospinning process, most importantly, the necessity for a high intensity electric field and the conductivity requirement for the polymer solution.

## Figures and Tables

**Table 1:** Fiber size and morphology of PVP fibers spun from a 22% w/w solution at various rotational speeds.

Speed (rpm)	Fiber Diameter (μm)	Beading
3500	4.38 ± 2.60	yes
4500	3.82 ± 2.42	yes
5500	1.40 ± 0.75	no
6500	2.87 ± 1.05	no
7500	2.55 ± 1.27	no

**Table 2:** Lap content of the fiber mat samples.

Sample	Measured LB Content (%)	Theoretical LB Content (%)	Relative Content (%)	Average Relative Content (%)	Standard Deviation
Lap-PVP_1	12.07		102.59		
Lap-PVP_2	11.68	11.76	99.27	100.99	1.66
Lap-PVP_3	11.89		101.11		

## Data Availability

The data presented in this study are available on request from the corresponding author.
